# Hemorrhagic Transformation in Acute Ischemic Stroke: A Quantitative Systematic Review

**DOI:** 10.3390/jcm11051162

**Published:** 2022-02-22

**Authors:** Asaf Honig, Jennifer Percy, Amir A. Sepehry, Alejandra G. Gomez, Thalia S. Field, Oscar R. Benavente

**Affiliations:** 1 Division of Neurology, Vancouver Stroke Program, University of British Columbia, Vancouver, BC V6T 2B5, Canada; jenniferpercy7@gmail.com (J.P.); sepehryaa@gmail.com (A.A.S.); alejandra.gomez.glez@gmail.com (A.G.G.); thalia.field@ubc.ca (T.S.F.); oscar.benavente@ubc.ca (O.R.B.); 2 Department of Neurology, Hadassah-Hebrew University Medical Center, Jerusalem 91120, Israel; 3 Clinical Psychology Program, Adler University, Vancouver, BC V6B 3J5, Canada

**Keywords:** hemorrhagic transformation, intravenous thrombolysis, ischemic stroke, parenchymal hematoma

## Abstract

The prevalence and risk factors of hemorrhagic transformation (HT) after acute ischemic stroke HT have not been adequately delineated. We performed a systematic review and meta-analysis to identify English-language prospective observational MEDLINE and EMBASE-listed reports of acute ischemic stroke with HT published from 1985–2017. Studies that used the ECASS-2 definitions of hemorrhagic transformation subtypes, hemorrhagic infarction (HI), and parenchymal hematoma (PH) were included. Patients treated with intravenous thrombolysis with tissue plasminogen activator (IV-tPA) were compared with those who did not receive thrombolysis. A total of 65 studies with 17,259 patients met inclusion criteria. Overall, HT prevalence was 27%; 32% in patients receiving IV-tPA vs. 20% in those without. Overall PH prevalence was 9%; 12% in IV-tPA treated patients vs. 5% in those without. HT was associated with a history of atrial fibrillation (OR 2.94) and use of anticoagulants (OR 2.47). HT patients had higher NIHSS (Hedge’s-G 0.96) and larger infarct volume (diffusion-weighted MRI, Hedge’s-G 0.8). In IV-tPA treated patients, PH correlated with antiplatelet (OR 3) and statin treatment (OR 4). HT (OR 3) and PH (OR 8) were associated with a poor outcome at 90-day (mRS 5–6). Hemorrhagic transformation is a frequent complication of acute ischemic stroke and is associated with poor outcome. Recognition of risk factors for HT and PH may reduce their incidence and severity.

## 1. Introduction

Hemorrhagic transformation (HT) occurs frequently in patients with acute ischemic stroke. While HT may be part of the natural disintegration process of the infarcted tissue [1], it is unclear to what extent it may exert its own deleterious effect independent of those attributed to the infarcted tissue [2].

There are two accepted classification schemes for HT assessment; one is based strictly on radiological criteria, while the second combines both clinical and radiological variables. The former was used as a safety end point for intravenous thrombolysis with tissue plasminogen activator (IV-tPA)-related HT in the European Cooperative Acute Stroke Trials (ECASS) [3,4,5]. The ECASS classification categories are no HT, HT into hemorrhagic infarction (HI), manifesting as small petechial hemorrhage along the margins of the infarct, and parenchymal hematoma (PH), manifesting as confluent hematoma. HI and PH are subdivided into type 1 and 2 for the milder and more severe forms, respectively (without HT, HI, PH, and PH2). Notably, PH2, the most severe form of HT, is classified as blood clots exceeding 30% of the infarct area with significant space-occupying effect. In cases of HT classed as ECASS-I, PH2 was associated with both neurological deterioration (OR 32.3) and increased 3-month mortality (OR 18) [4] compared with patients without PH2.

The second HT classification system is based on clinical presentation; (a) symptomatic vs. (b) asymptomatic. Unfortunately, different definitions of symptomatic HT have been adopted, introducing biases regarding prevalence, risk factors, and prognosis [5,6]. ECASS-III required concurrent hemorrhage on CT scan and neurological deterioration, with an increase of >4 points in the National Institutes of Health Stroke Score (NIHSS) to diagnose symptomatic HT [3]. Subdividing HT into symptomatic vs. asymptomatic is problematic because it is difficult to attribute neurological deterioration solely to HT. Furthermore, the effect of asymptomatic HT on outcome remains controversial [7,8].

We aimed to characterize the prevalence, risk factors, and prognosis of HT in patients with acute ischemic stroke by conducting a systematic review of studies that reported HT. As rates of HT are higher in patients treated with IV-tPA [9], we specifically investigated interactions between possible predictors of HT in IV-tPA treated and untreated subpopulations. Additionally, we examined rates of HT subtypes in patients of East-Asian ethnicity, since they are more prone to suffer from HT [8,10].

## 2. Materials and Methods

This study was registered with the International Prospective Register of Systematic Reviews; PROSPERO 2017 (Registration number CRD42017074806) and adheres to the PRISMA guidelines for preferred reporting in meta-analyses [11]. Two authors (A.H. and J.P.) identified potentially relevant studies and independently extracted data. For purposes of quality assurance, a third author (O.R.B.) extracted information from ten studies and validated the results.

### 2.1. Study Identification and Classification

An electronic search of the MEDLINE and EMBASE Ovid (Wolters Kluwer, Alphen aan den Rijn, The Netherlands) databases for English-language prospective observational studies published between January 1985 and May 2017 that reported rates of HT using the search terms “cerebral infarction/or brain isch*emia or stroke” and “h*emorrhagic transformation.” Studies were included if they recorded baseline and follow-up brain imaging (CT or MRI) demonstrating hemorrhagic transformation in an adult population and used the ECASS definition of HT; conference abstracts or letters to the editor, reviews, case series with fewer than 10 patients, and retrospective studies (Supplemental Material) were excluded.

### 2.2. Each Study Was Classified According to the Following Criteria

IV-tPA treatment—According to the study protocol, each study population was classified as either IV-tPA treated or untreated. In a study including both subpopulations, data was extracted for each subgroup separately. Data from mixed populations of IV-tPA treated and untreated patients that did not differentiate the two subpopulations were only used for the total population included in the global assessment of each factor. Similarly, data from patients who underwent any endovascular procedure was included in the global assessment of each factor and not included in the IV-tPA treated and untreated analysis.

Imaging timing and modality—To assess the effect of imaging modality on the frequency of HT, studies were categorized based on whether CT or MRI was used in repeat scan after admission. Studies that included both imaging modalities were excluded from this specific analysis. To assess HT frequency with respect to timing of repeat imaging, studies were divided into series where the repeat scan after the index event was performed within 72 h or after a longer interval.

Ethnicity—Studies published by an institution in an East-Asian country that included only the local population were designated as such and were compared to studies from non-East-Asian countries.

### 2.3. Data Extraction

For each study, demographic, clinical, and radiological data were extracted in accordance with PRISMA criteria [11]. Using predefined variables, two investigators (A.H. and J.P.) independently evaluated all studies and extracted the data. Data were collected by treatment (IV-tPA treated versus untreated patients) and according to the ECASS radiological classification (without HT, HI, PH, and PH2). In studies where results were given for a mixed population of IV-tPA treated and untreated patients, the available data were used only when calculating the effect of a certain variable on the total patient population. Supplemental Table S1 lists all studies included in the meta-analysis, and Supplemental Tables S3–S5 detail the specific studies used in each sub-analysis. For the sake of brevity, some of the studies used in the analysis may not be cited in the body of this paper.

### 2.4. Data Analysis

Two types of aggregate effect-size estimates, odd ratios (OR), and Hedges’ g (HG), were calculated using continuous and categorical data. Categorical data were used to create the contingency table of analysis for OR (measure of association), and continuous data (i.e., mean, standard deviation, and sample size) were used to generate HG, a measure of group differences. The standard practice for interpretation of HG, as per Cohen’s suggestions [12], is to stratify findings as a small effect (HG < 0.2), a medium effect (HG 0.2−0.8), or a large effect (HG > 0.8).

Categorical variables included imaging modality (CT, MRI) and repeat image time (≤72, >72 h), ethnicity (East Asian, non-East Asian), gender, previous medical condition (Yes/No), hypertension (HTN), diabetes mellitus (DM), atrial fibrillation (AF), hyperlipidemia, and history of alcohol consumption. Treatments prior to acute stroke included anticoagulation, antiplatelets, and statins. Functional outcome was measured at 90 days by modified Rankin score (mRS) of 0–1, 2−4, and 5–6.

Continuous variables included age, low density lipoprotein (LDL) cholesterol level, admission glucose level (AGL), systolic and diastolic blood pressure, NIHSS score, and infarct volume measured on diffusion-weighted MRI (DWI).

Neuroradiological markers, patient body temperature, and renal impairment data were analyzed qualitatively.

For each variable, three group comparisons were made: HT vs. no HT, PH vs. no PH, and PH vs. HI. Data analysis was performed for IV-tPA-treated and untreated patients separately whenever possible.

Statistical analyses were carried out using the Comprehensive Meta-analysis software (Verson 2.0, Biostat, Englewood, NJ, USA) [13] including the random effect model and analysis of continuous and categorical data to report the aggregate prevalence estimate and the association markers with 95% confidence intervals (CI) for all variables. We examined for the presence of between-studies heterogeneity in the results by relying on the magnitude of the I-square statistics [14].

### 2.5. Assessment of the Risk of Bias

Two reviewers (A.H. and J.P.) independently assessed the risk of bias for individual studies according to widely accepted tools [15], noting methodology for participant selection, HT outcomes, blinding, loss to follow-up, methods for controlling confounding, and declaration of conflicts of interest.

## 3. Results

**Figure 1:**
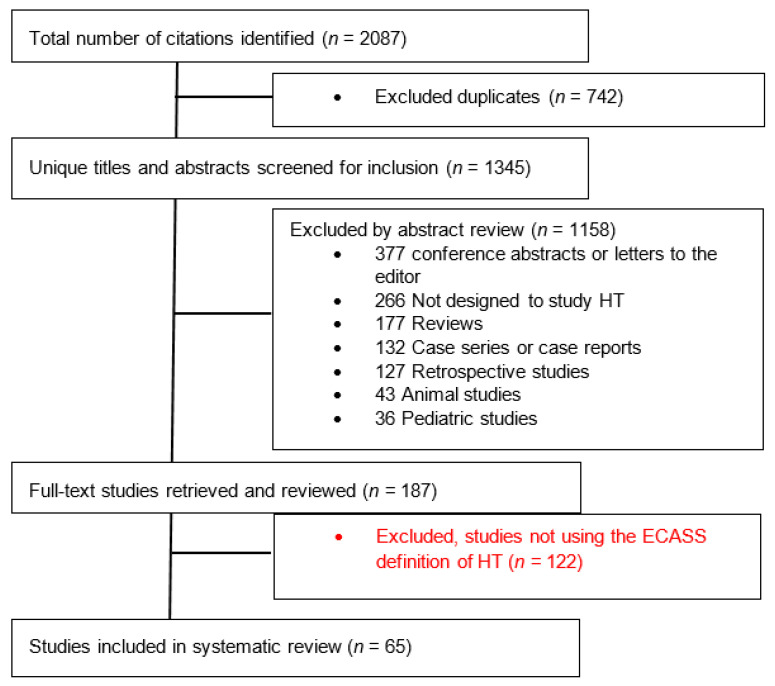
Flow diagram of the study selection process. HT—Hemorrhagic Transformation; ECASS—European Cooperative Acute Stroke Study.

A total of 2087 studies were identified; 65 studies (seven randomized clinical trials and 58 observational studies) enrolling a total of 17,259 patients met selection criteria and were included in this analysis (Figure 1). The characteristics of all included studies are summarized in Supplementary Tables S1 and S2.

### 3.1. Hemorrhagic Transformation

The aggregated rates of HT are summarized in Table 1. Overall prevalence of HT was 27% (95% CI 23–30%). Patients treated with IV-tPA had HT prevalence of 32% (95% CI 27–37) in contrast to 20% (95% CI 14–27) in the untreated group (OD 1.9; CI 1.7–2.1, *p* < 0.001). More importantly, IV-tPA treated patients, compared with IV-tPA untreated patients, had significantly higher rates of both PH (12% versus 5%, OD 2.8, CI 2.3–3.4, *p* < 0.001) and PH2 (5% versus 3%, OD 2.1, CI 1.4–3.2, *p* < 0.0001). A more pronounced effect from IV-tPA treatment was observed in East-Asian patients for both PH (15% versus 4%, OD 2.3, CI 1.3–4.2, *p* = 0.004) and PH2 (9% versus 2%, OD 12.5, CI 5.8–26.7, *p* < 0.0001).

Similar rates of PH were diagnosed using either CT or MRI (9% for both). In contrast, higher rates of HI were diagnosed using MRI (20% versus 15%).

Table S3 details which studies were included in each analysis.

### 3.2. Demographics

Age—Overall, 15 studies including 3480 patients were included in analysis of patient age [16,17,18,19,20,21,22,23,24,25,26,27,28,29,30] (Table 2). Between group comparison showed that for the full sample, patients with HT were significantly older than those without HT (HG 0.13, CI 0.05–0.2) and patients with PH were older compared to patients without PH (HG 0.22, CI 0.09–0.34). In the IV-tPA analysis, treated patients with PH were significantly older than those without PH (HG 0.27, CI 0.04–0.5); however, there was no significant difference for untreated patients with vs. without PH (HG 0.12, CI −0.52–0.76).

Male sex was provided in 18 studies including 5809 patients; 3312 (57%) were male [7,17,19,21,22,23,24,25,26,28,29,30,31,32,33,34,35,36] (Table 2). In the overall population male sex was not associated with a higher frequency of HT; however, it was associated with PH (OR 1.5, CI 1.07–2.11).

### 3.3. Baseline Comorbidities

Details regarding underlying chronic HTN were provided in 14 studies including 4743 patients; 2732 (58%) had chronic HTN [7,21,23,25,27,29,33,35,36,37,38,39,40,41] AH VERIFY (Table 2). While chronic HTN was not associated with HT in the full sample (OR 1.2, CI 0.9–1.7), it was associated with PH in IV-tPA treated patients (OR 1.51, CI 1.1–2.07).

The frequency of DM was given in 21 studies including 7037 patients; 1657 (24%) had DM [7,16,18,19,21,22,23,24,25,26,27,28,29,31,33,35,36,39,41,42,43] (Table 2). There was a tendency towards an association of DM with HT that did not reach significance (OR 1.23, CI 0.97–1.56); however, DM was more strongly associated with PH compared with HI (OR 1.66, 1.05–2.61) in a mixed population of IV-tPA treated and untreated patients.

Details regarding hyperlipidemia were given in 10 studies including 2619 patients (positive in 630, 24%) [19,21,22,26,27,32,35,39,41,42] (Table 2). The presence of hyperlipidemia was negatively associated with HT in IV-tPA untreated patients (OR 0.53, CI 0.31–0.91).

LDL levels upon admission were available in five studies including 2055 patients [20,23,36,39,43] (Table 2). HT patients had lower LDL levels compared with patients without HT (HG −0.3, CI −0.12– −0.48); however, a similar association was not found with PH.

Details regarding alcohol abuse was provided in four studies including 2289 patients, of whom 284 (12%) had a history of abuse [27,33,36,43] (Table 2). There was no association with either HT (OR 1.32, 0.92–1.88) nor PH (OR 1.02, 0.5–2.08) in a mixed population of IV-tPA treated and untreated patients in this sample.

### 3.4. Chronic Treatment

Information regarding chronic anticoagulation treatment was provided in four studies including 2470 patients, of whom 126 (5.1%) were under treatment [20,26,36,43] (Table 2). In the overall population anticoagulation was clearly associated with HT (OR 2.47, CI 1.64–3.72).

Details regarding chronic antiplatelet therapy were provided in eight studies including 4464 patients, of whom 1312 (29%) were under treatment [19,20,25,26,28,36,43,44] (Table 2). Antiplatelet treatment was associated with PH in the overall population (OR 2.25, CI 1.26–4.02) and to a larger extent in IV-tPA treated patients (OR 3.15, CI 1.39–7.17).

The frequency of chronic statin treatment was given in six studies including 2734 patients; 420 of them (15%) were under treatment [23,26,28,36,43,45] (Table 2). Statin treatment tended to be associated with PH in the overall population (OR 2.15, CI 0.98–4.76) and significantly associated with PH in IV-tPA treated patients (OR 3.58, CI 1.41–9.05), with the caveat that only two studies were included in the IV-tPA subanalysis.

### 3.5. Clinical Data upon ER Admission

Potential association with systolic and diastolic BP, body temperature, and glucose levels was examined (Table 3).

Data for systolic BP upon admission were available from 12 studies including 2582 patients [16,17,18,21,22,24,26,32,35,39,42,43]. Systolic pressure was higher in patients with PH versus those without in the overall population (HG 0.22, CI 0–0.45) and more specifically in IV-tPA treated patients (HG 0.34, CI 0.11–0.57).

Details regarding diastolic BP upon admission were also provided in 12 studies including 2631 patients [16,17,18,21,22,24,26,32,35,39,42,43]. No between-group differences were found for diastolic BP on any comparison made. One study [42] found that greater diastolic BP variability was associated with higher rates of HT, independent of mean values.

Information regarding glucose level upon admission was given in 13 studies including 3845 patients [16,17,18,21,22,23,24,26,35,36,39,42,43]. Between-group comparisons showed higher glucose levels in HT patients compared to those without HT (HG 0.19, CI 0.09–0.29) and in PH patients compared with patients without PH (HG 0.31, CI 0.15–0.48). In a comparison of the two HT subtypes, PH patients had higher glucose levels than HI patients (HG 0.42, CI 0.16–0.67).

An association between body temperature and HT by ECASS classification was found in four studies; however, we were not able to aggregate the data for meta-analysis. One study [27] found that a higher body temperature in the first day post-stroke was associated with HT (OR 7.3, CI 2.4–22.6) in non-IV-tPA treated patients. One randomized clinical trial [46] compared outcomes with therapeutic hypothermia in patients who had a middle cerebral artery (MCA) clot recanalized by stent thrombectomy and/or IV-tPA, and found lower rates of HT in (14% versus 39%, *p* = 0.016) and less cerebral edema (no cerebral edema in 17% with treatment versus 54% without, *p* = 0.001). Two studies of patients with MCA occlusion treated with IV-tPA [40,47] examining the association of PH with increased body temperature found a trend that almost reached statistical significance [47] in the larger cohort but was not significant in the second study [40].

### 3.6. Stroke Mechanism

Overall rates of HT were substantially different in comparisons of cases with different proposed etiologies.

The prevalence of AF was assessed in 15 studies including 4982 patients, with AF in 1176 (24%) [7,16,19,20,22,24,25,27,29,31,32,33,36,39,41] (Table 2). There was an association between AF and both HT (OR 2.86, 1.86–4. 37) and PH (OR 2.25, 1.47–3.44).

Lacunar etiology was associated with extremely low rates of HT in seven studies [8,24,33,48,49,50,51]. AH VERIFY.

### 3.7. Stroke Severity

Stroke severity was assessed by both NIHSS score and DWI volume upon admission. Admission NIHSS was available in six studies including 1202 patients [19,20,23,24,30,31]. HT patients had remarkably higher admission NIHSS compared with patients without HT in general (HG 0.96, CI 0.48–1.45), and in the subpopulation of IV-tPA treated patients in particular (HG 0.93, CI 0.36–1.51). Similarly, PH patients had higher admission NIHSS than non-PH patients (HG 0.45, CI 0.22–0.69).

The DWI ischemic volume was available in five studies including 656 patients [16,20,23,24,31]. DWI ischemic volumes were larger on MRI studies performed on the first day of ictus in HT compared with non-HT patients (HG 0.83, CI 0.1–1.76). The aggregated data did not allow further subgroup analysis; however, one study [23] found that the severity of hypoperfusion on MRI perfusion studies is more predictive of HT than DWI volume. Similarly, three CT perfusion studies [41,52,53] found a direct strong correlation between hypoperfusion and HT.

Several radiological and laboratory biomarkers suggesting blood–brain barrier (BBB) disruption were reported in selected studies. Unfortunately, there were not sufficient data for any marker to enable meta-analysis. Neuroimaging signs of BBB disruption found on dual-energy CT studies performed following stent thrombosis found a strong association with HT (OR 4.5, 1.2–16.4) [54] and parenchymal enhancement on postcontrast T1-weighted MRI following IV-tPA were predictive and localized subsequent HT in one study [55]. Laboratory biomarkers suggesting BBB disruption in IV-tPA treated patients were found to be strong independent predictors of PH in multivariate analysis, including the neutrophil-to-lymphocyte ratio (OR 8.5, CI 2.7–26.9 for a ratio >10.6) [37], baseline matrix-metalloproteinase-9 (OR 9.6, CI 1.3–70.3) [56], and platelet-derived growth factor C (PDGFC), where a level >175 predicted PH with 90% sensitivity [47].

### 3.8. Outcomes

The 90-day mRS was available in four studies that included 1838 patients. Among them, 388 patients (21%) had an mRS of 5–6 at 90 days [7,25,31,57] (Table 4). This high mRS was associated with HT (OR 2.16, 1.7–2.75), and even more strongly associated with PH (OR 5.4, CI 3.2–9.1) in the overall group. When the association with a poor outcome was analyzed the subgroup of IV-tPA treated patients, there was a clear association with HT (OR 2.22, CI 1.7–2.92) and a stronger association with PH (6.25, CI 3.2–12.3). Conversely, in those who did not receive IV-tPA, neither HT nor PH were associated with a poor outcome. In contrast, based on data from three studies including 1197 patients, a 90-day mRS of 0–1 found in 434 patients (36%) [7,31,58] was less frequent in those with HT (OR 0.45, CI 0.28–0.71) and even less likely in those with PH (OR 0.35, CI 0.15–0.86).

## 4. Discussion

Aggregated data in this meta-analysis revealed that IV-tPA-treated patients had higher rates of HT (32% versus 20%), PH (12% versus 5%), and PH2 (5% versus 3%) compared to those who did not undergo thrombolysis (*p* < 0.001 for all conditions). This finding is in agreement with a meta-analysis of individual patient of data from nine random control trials that found that IV-tPA significantly increased the odds of PH2 (OR 5.55, 95% CI 4.01–7.70, *p* < 0.0001) (6.8% versus 1.3%) [9]. Interestingly, IV-tPA-associated HT was more pronounced in the East-Asian patients, who showed higher rates of IV-tPA associated PH and PH2 compared to non-East-Asians (15% versus 12% and 9% versus 5%, respectively). This increased tendency of East-Asian populations to show higher rates of HT in response to IV-tPA treatment was also reflected in two randomized control trials in Japan and China [8,10], which showed higher rates of severe HT in the regular versus reduced IV-tPA dosage. Moreover, a large observational study found that a low-dose alteplase strategy was comparable to the standard-dose treatment in terms of effectiveness and safety [59].

Similar to previously reported literature [60], our results show that both CT and MRI have similar rates of PH in general and PH2 in particular, thus, allowing us to aggregate patients with PH seen on different imaging modalities without introducing a significant bias.

Understanding the underlying pathophysiological processes that lead to HT may give a plausible explanation to the different variables found to be associated with HT in general and with PH in particular. These processes include ischemia-induced metabolic changes, which, together with an inflammatory response [61] lead to BBB disruption [62]. BBB disruption, along with impairment of cerebral vasculature autoregulatory control, predisposes to blood extravasation when the ischemic tissue is eventually reperfused [63], resulting in HT [64]. Moreover, Matrix metalloproteinase (MMP) expression is related to blood–brain barrier disruption after cerebral ischemia in both IV-tPA treated and untreated ischemic stroke patients [65,66]. In addition, previous systematic reviews performed separately on IV-tPA treated and untreated patient populations, found a clear association between the size of the ischemic territory and prevalence of HT [67].

Among IV-tPA treated patients, PH patients were significantly older than patients without PH (HG 0.27, CI 0.04–0.5) However, this was not found in untreated IV-tPA patients (HG 0.12, CI −0.52–0.76). This difference may be because elderly patients could be more readily susceptible to BBB permeability under ischemia [68] and have a higher burden of cerebral microbleeds (CMBs) [69,70]. A systematic review found that in IV-tPA treated patients, the presence of CMBs increases the risk of symptomatic HT [71].

Male gender was not found to be associated with HT in general, but was associated with PH (OR 1.5, CI 1.07–2.11). One possible explanation could be the independently higher burden of cerebral microbleeds (CMBs) in men (OR 1.7, CI 1.3–2.3) [71].

Chronic hypertension was associated with PH only in the IV-tPA treated patients (OR 1.51, CI 1.1–2–07), possibly due to longstanding hypertension-induced vasculopathic changes such as Charcot microaneurysms and CMBs [72].

DM tended to be associated with HT (OR 1.23, CI 0.97–1.56) and was significantly associated with having PH rather than HI (OR 1.66, 1.05–2.61). Again, it may be attributed to higher burden of small vessel disease (SVD) in diabetic patients. Alternatively, higher glucose levels in diabetic patients may be associated with enhanced bleeding [73]. We found higher acquired generalized lipodystrophy (AGL) in comparisons of HT versus non-HT patients (HG 0.19, CI 0.09–0.3), and PH versus non-PH patients (HG 0.31, CI 0.15–0.48). In an adjusted meta-analysis of studies of IV-tPA-treated patients, higher AGL was associated with more symptomatic intracranial hemorrhage [73]. One possible mechanism is increased BBB disruption [74], which increases blood extravasation into the infarcted brain parenchyma.

Hyperlipidemia was negatively associated with HT in IV-tPA untreated patients. This seemingly protective effect was strong when both LDL level (HG −0.66, CI −0.29–1) and history of dyslipidemia (OR 0.53, CI 0.31–0.93) were examined separately. As seen with the known negative association between spontaneous intracerebral hemorrhage (ICH) and LDL level [75], it is unclear whether LDL itself plays a protective role or whether it is, instead, a biomarker for other mechanisms. A previous study found a body-mass index (BMI) >25 has an independent protective effect against HT (OR 0.39, 0.17–0.87) [76]. This paradoxical effect of obesity has been attributed to previous reports on higher levels of multiple coagulation factors [77] and a suboptimal response to antiplatelet medications [78,79] in obese patients.

Chronic medications play a significant role in the pathophysiology of HT [80]. The finding that previous treatment with anticoagulation increased risk for HT in general and PH in particular seems natural. Interestingly, chronic antiplatelet use was not associated with HT (OR 1, CI 0.83–1.23) but was associated with PH (OR 2.25, CI 1.26–4). We suggest that once bleeding occurs within the brain parenchyma, the ongoing disruption of platelet function facilitates the bleeding process. We presume the ongoing effect of antiplatelet treatment is further intensified in IV-tPA-treated patients, where there was a higher risk of PH (OR 3.15, CI 1.4–7.2). Our findings are in agreement with a large systematic review including 108,588 patients [81], which found that patients receiving long-term antiplatelet medications were associated with greater risks of developing symptomatic intracranial hemorrhage after IV-tPA. Surprisingly, a similar association was found for pretreatment with statins with PH in IV-tPA-treated patients (OR 3.58, CI 1.4–9). Our findings on this point are in contrast with previous large cohorts that did not show independently increased rates of symptomatic HT in IV-tPA-treated patients with a history of statin treatment [82,83]. Statin treated patients may have long-standing atherosclerosis-inducted arteriopathy that could be prone to bleeding. Unfortunately, our inability to adjust for individualized patient data hampers additional investigation of this finding.

Emergency room information is essential for HT risk assessment. Among IV-tPA-related patients, the HT group had higher systolic blood pressure (BP) values compared with those without HT (HG 0.34, CI 0.11–0.57). These differences were not found in patients who did not undergo thrombolysis. The association between higher systolic BP values and symptomatic HT was reported previously [84]. The underlying pathophysiology may include higher perfusion to vessels that lack autoregulation in the setting of IV-tPA. The role of BP in the acute management of patients without IV-tPA treatment deserves further investigation. We found qualitatively that higher temperature increases HT. Animal models have shown that hyperthermia increases BBB permeability, and consequently brain edema, in addition to increasing the fibrinolytic activity of IV-TPA [85].

Stroke severity, as assessed by the NIHSS, showed the highest degree of association with HT (HG 0.96, CI 0.48–1.45), but its association with PH was weaker (HG 0.45, CI 0.22–0.69). Similar findings were found for associations for ischemic volume DWI with HT (HG 0.76, CI 0.02–1.5) and with PH (HG 0.35, CI 0.07–0.62) suggesting that other predictors may play a significant role in PH occurrence.

Stroke mechanism impacts the rate of HT. AF was associated with HT and PH while large vessel atherosclerosis (LVA) was not. This finding is in agreement with a previous meta-analysis of IV-tPA treated patients [86]. The association for AF with PH could be attributed to higher rates of pretreatment with anticoagulation and a larger infarcted territory [87]. However, after adjustment for infarct size, several studies have shown the independent role of a cardioembolic mechanism for HT [57,82,88], and an even higher association with PH [36]. Perhaps the brain parenchyma in patients with LVA has been exposed to ongoing ischemia, leading to collateral flow buildup, and, therefore, is less subject to HT in contrast to abrupt occlusion and possibly recanalization, as seen in cardioembolic etiology.

In regard to functional outcome, PH was strongly associated with a poor outcome (mRS 5–6) in IV-tPA-treated patients (OR 6.25, CI 3.2–12.3), but not in patients who had no IV thrombolysis. Perhaps the pathophysiological mechanisms underlying PH differ between IV-tPA-treated and untreated patients; HT in the non-IV-tPA treated patients is a more natural process of disintegration of the ischemic tissue and takes place in a more gradual way. One large (n = 954 patients) observational study on IV-tPA treated [7] patients highlighted the independent deleterious effect of HT on prognosis.

Our work has several limitations. First, since we lack individual patient data, we can only show the aggregate of the available data and cannot adjust it for possible confounders. Second, HT was assessed in different timeframes and using different imaging modalities, thereby introducing a possible bias. Third, we did not assess the risk of bias for each of the studies included using the Newcastle–Ottawa assessment scale. This was an intentional choice to enable us to include studies from many international locations and that enrolled patients of diverse ethnicities. However, our work also has several possible strengths. It uses unified criteria to assess HT, it is up to date with the current literature and is of large scale, thereby minimizing the risk of bias. We hope this may allow the clinician to determine the likelihood of this event based on an individual patient’s pre-stroke historical characteristics and initial clinical, laboratory, and imaging evaluations in the emergency department.

## 5. Conclusions

Hemorrhagic transformation is a frequent complication of acute ischemic stroke and is associated with poor outcome. Recognition of risk factors for HT and PH may reduce their incidence and severity.

Prospective studies to further characterize these variables in a longitudinal manner are warranted.

## Figures and Tables

**Table 1:** Prevalence (%) of hemorrhagic transformation.

		Rates of Hemorrhagic Transformation		Rates of Hemorrhagic Infarction (HI 1 + 2)		Rates of Parenchymal Hematoma (PH 1 + 2)		Rates of PH2	
		W/out tPA Prevalence% (95% CI)	With tPA Prevalence% (95 CI)	W/out tPA Prevalence% (95% CI)	With tPA Prevalence% (95 CI)	W/out tPA Prevalence% (95% CI)	With tPA Prevalence% (95 CI)	W/out tPA Prevalence% (95% CI)	With tPA Prevalence% (95 CI)
**General Frequencies**		**20%**	**32%**	**14%**	**18%**	**5%**	**12%**	**3%**	**5%**
		(14–27)	(27–37)	(9–21)	(15–22)	(4–7)	(10–15)	(1–5)	(4–7)
		624/3824	1754/6442	473/3824	1094/6442	151/3824	701/6748	29/1325	174/3825
**Ethnicity** **East-Asians**		**15%**	**35%**	**11%**	**16%**	**4%**	**15%**	**2%**	**9%**
		(12–19)	(19–56)	(8–15)	(9–26)	(1–13)	(7–30)	(0.1–27)	(2–39)
		51/340	344/1645	38/340	216/1645	13/340	156/1834	9/340	36/142
**Non-East Asians**		**21%**	**31%**	**14%**	**19%**	**5%**	**12%**	**3%**	**5%**
		(14–29)	(27–36)	(9–22)	(16–23)	(4–8)	(10–14)	(1–5)	(4–7)
		573/3484	1410/4797	435/3484	878/4797	138/3484	545/4914	20/985	138/2783
**Imaging modality**	**CT**	26%		15%		9%			
		(21–31)		(12–19)		(8–12)			
		2197/9899		1436/9899		802/10,205			
	**MRI**	31%		20%		9%			
		(24–39)		(15–25)		(7–13)			
		989/4553		641/4553		348/4553			
**Timing of imaging**	**<72 h**	29%		18%		10%			
		(26–33)		(16–21)		(8–12)			
		2831/10,439		1876/10,439		996/10,745			
	**>72 h**	20%		12%		7%			
		(14−28)		(9−17)		(4−10)			
		698/5197		439/5197		259/5197			

HT: Hemorrhagic Transformation; HI: Hemorrhagic Infarction; PH: Parenchymal Hematoma. Rates marked in bold indicate a statistically significant difference between IV-tPA treated and untreated patients.

**Table 2:** Patient Group Differences based on risk factors; effect size (95% CI); heterogeneity magnitude I2.

Variable		HT vs. Non-HT	PH vs. Non-PH	PH vs. HI
Baseline patients’ characteristics				
**Age** **HG**	W/O tPA	0.1 (−0.124–0.325) (*p* = 0.3791); 0	NG	NG
	With tPA	0.3 (−0.249–0.849) (*p* = 0.1181); 0	**0.271** (0.037–0.505) (*p* = 0.0233); 16.7 5 studies included 102 vs. 552	NG
	Total pts. included	**0.126** (0.048–0.202) (*p* = 0.0017); 0	**0.215** (0.087–0.344) (*p* = 0.0012); 0	**0.267** (0.084–0.45) (*p* = 0.004)
		15 Studies included	10 studies included	5 studies included
		968 vs. 2512	273 vs. 1729	161 vs. 386
**Male** **OR**	W/O tPA	1.71 (0.55–5.33) (*p* = 0.3575); 68.3 3 studies included 36/64 vs. 166/304	NG	NG
	With tPA	0.89 (0.7–1.14) (*p* = 0.3575); 0	1.37 (0.78–2.40) (*p* = 0.2709); 58.6	1.08 (0.61–1.90) (*p* = 0.7956)
		5 studies included	7 studies included	2 studies included
		195/366 vs. 511/910	137/213 vs. 825/1475	60/108 vs. 105/199
	Total pts. included	1.07 (0.88–1.31) (*p* = 0.4734); 42.4	**1.50 (1.07–2.11) (*p* = 0.0180); 50.3**	**1.43 (1–2) (*p* = 0.0476); 24.7**
		18 studies included	14 studies included	9 studies included
		744/1282 vs. 2568/4527	281/431 vs. 2395/4263	204/326 vs. 352/640
**Chronic** **hypertension** **OR**	W/O tPA	1.67 (0.52–5.32) (*p* = 0.3842); 83.4	1.74 (0.54–5.54) (*p* = 0.3513)	2.69 (0.85–8.53) (*p* = 0.0928); 0
		4 studies included	2 studies included	3 studies included
		102/145 vs. 261/440	18/22 vs. 224/310	22/26 vs. 50/82
	With tPA	1.74 (0.85–3.55) (*p* = 0.5851)	**1.51 (1.10–2.07) (*p* = 0.0101); 0**	1.54 (0.96–2.48) (*p* = 0.0743)
		2 studies included	6 studies included	2 studies included
		161/307 vs. 406/787	124/194 vs. 745/1414	64/108 vs. 97/199
	Total pts. included	1.21 (0.86–1.71) (*p* = 0.2685); 75	**1.29 (1.04–1.61) (*p* = 0.0226); 0**	1.31 (0.87–1.96) (*p* = 0.1961); 35.7
		11 studies included	13 studies included	10 studies included
		562/996 vs. 2170/3485	248/392 vs. 2665/4350	192/310 vs. 340/649
**Diabetes** **OR**	W/O tPA	1.41 (0.66–3.03) (*p* = 0.3755); 70.3 5 studies included 61/156 vs. 169/544	NG	2.4 (0.96–6.02) (*p* = 0.0616); 0 3 studies included 13/26 vs. 23/82
	With tPA	0.97 (0.69–1.38) (*p* = 0.8781); 0 4 studies included 53/340 vs. 136/849	NG	1 (0.1–9.91) (*p* = 0.9975) 2 studies included 23/108 vs. 25/199
	Total pts. included	1.23 (0.97–1.56) (*p* = 0.0892); 51.9 21 studies included 339/1466 vs. 1318/5571	NG	**1.66 (1.05–2.61) (*p* = 0.0287); 35.2** 11 studies included 83/323 vs. 117/680
**Hyperlipidemia** **OR**	W/O tPA	**0.53 (0.31–0.93) (*p* = 0.0268); 0**	0.81 (0.29–2.26) (*p* = 0.6833)	1.14 (0.37–3.46) (*p* = 0.5234); 0
		**3 studies included**	2 studies included	3 studies included
		**20/109 vs. 84/276**	5/22 vs. 82/310	5/26 vs. 14/82
	With tPA	0.52 (0.25–1.06) (*p* = 0.0739) 2 studies included 13/59 vs. 44/125	1.25 (0.61–2.58) (*p* = 0.5406); 57.7 5 studies included 50/102 vs. 222/552	NG
	Total pts. included	0.8 (0.62–1.03) (*p* = 0.0878); 0.7	1.02 (0.57–1.84) (*p* = 0.9408); 46.6	0.76 (0.33–1.75) (*p* = 0.8224); 0
		10 studies included	8 studies included	5 studies included
		101/452 vs. 529/2167	56/138 vs. 338/980	9/56 vs. 29/136
**Alcohol** **Abuse** **OR**	W/O tPA	1.47 (0.55–3.9) (*p* = 0.4389)	1.97 (0.39–10.05) (*p* = 0.4146)	1.62 (0.28–9.56) (*p* = 0.5912)
	With tPA	NG	NG	NG
	Total pts. included	1.32 (0.92–1.88) (*p* = 0.1295)	1.02 (0.5–2.08) (*p* = 0.9458)	1.15 (0.49–2.72) (*p* = 0.7435)
		4 studies included	3 studies included	3 studies included
		56/342 vs. 228/1947	10/78 vs. 225/1960	10/78 vs. 20/165
**Prev.** **anticoagulation** **OR**	W/O tPA	NG	NG	NG
	With tPA	NG	NG	NG
	Total pts. included	**2.47 (1.64–3.72) (*p* = 0.0000); 0**	**2.9 (1–8.55) (*p* = 0.0536)**	1.81 (0.42–7.74) (*p* = 0.4220)
		4 studies included	1 study included	1 study included
		40/378 vs. 86/2092	4/36 vs. 46/1089	4/36 vs. 4/62
**Prev.** **Antiplatelets** **OR**	W/O tPA	NG	NG	NG
	With tPA	NG	**3.15 (1.39–7.17) (*p* = 0.0061); 72.4** 4 studies included 61/165 vs. 237/1186	NG
	Total pts. included	1.1 (0.83–1.23) (*p* = 0.9313); 12.7	**2.25 (1.26–4.02) (*p* = 0.0063); 70**	1.38 (0.66–2.87) (*p* = 0.3959); 66.4
		8 studies included	6 studies included	3 studies included
		232/937 vs. 1080/3527	85/263 vs. 630/2822	50/190 vs. 78/414
**Prev. Statins** **OR**	W/O tPA	0.36 (0.45–1.29) (*p* = 0.2002)	NG	NG
	With tPA	NG	**3.58 (1.41–9.05) (*p* = 0.0071)** **2 studies included** **8/20 vs. 14/59**	NG
	Total pts. included	0.76 (0.45–1.29) (*p* = 0.3116); 53.1	2.15 (0.98–4.76) (*p* = 0.0576); 54.6	1.27 (0.2–8.15) (*p* = 0.7981)
		6 studies included	4 studies included	2 studies included
		54/440 vs. 366/2294	31/113 vs. 146/1404	10/68 vs. 11/103
**Atrial Fibrillation** **OR**	W/O tPA	**4 (1.00–15.92) (*p* = 0.049)**	5.1 (0.69–37.86) (*p* = 0.11)	**4.27 (1.13–16.23) (*p* = 0.033)**
	With tPA	1.98 (0.55–7.14) (*p* = 0.29)	1.26 (0.83–1.9) (*p* = 0.28)	0.75 (0.41–1.38) (*p* = 0.36)
	Total pts. included	**2.85 (1.86–4.38) (p < 0.0001)**	**2.25 (1.47–3.44) (*p* = 0.0002)**	**2.09 (1.26–3.48) (*p* = 0.004)**
		14 studies included	12 studies included	8 studies included
		381/1039 vs. 795/3943	146/359 vs. 942/4156	108/273 vs. 173/596
**LDL level** **HG**	W/O tPA	**−0.657 (−0.288–−1.03) (*p* = 0.0005)**	−0.089 (−0.673–0.496) (*p* = 0.2976)	0.234 (−0.442–0.909) (*p* = 0.4973)
	With tPA	NG	NG	NG
	Total pts. included	**−0.304 (−0.124–−0.483) (*p* = 0.0009)**	−0.075 (−0.304–0.155) (*p* = 0.5238)	0.018 (−0.259–0.296) (*p* = 0.8965)
		**5 studies included**	3 studies included	3 studies included
		**356 vs. 1699**	80 vs. 1400	80 vs. 128

NG: Not given; W/O: without. HT: Hemorrhagic Transformation; HI: Hemorrhagic Infarction; PH: Parenchymal Hematoma. HG: Hedges’ G. OR: Odds Ratio. Numbers marked in bold signify a statistically significant result.

**Table 3:** Patient data upon admission.

Variable		HT vs. Non-HT	PH vs. Non-PH	PH vs. HI
**Systolic BP**	W/O tPA	−0.101 (−0.407–0.202) (*p* = 0.5160)	−0.445 (−1.032–0.14) (*p* = 0.1360)	**−0.759 (−0.063–−1.454) (*p* = 0.0325)** **1 study included** **12 vs. 25**
	With tPA	0.05 (−0.188–0.285) (*p* = 0.6860)	**0.34 (0.11–0.569) (*p* = 0.0036)** **4 studies included** **89 vs. 448**	NG
	Total pts. included	0.043 (−0.056–0.141) (*p* = 0.3980)	**0.217 (−0.02–0.454) (*p* = 0.0722)**	−0.227 (−1.24–0.786) (*p* = 0.6605)
		12 studies included	6 studies included	2 studies included
		547 vs. 2035	116 vs. 689	27 vs. 57
**Diastolic BP**	W/O tPA	−0.213 (−0.519–0.094) (*p* = 0.1737)	−0.353 (−0.94–0.232) (*p* = 0.2371)	−0.483 (−1.166–0.2) (*p* = 0.1649)
	With tPA	0.08 (−0.15–0.312) (*p* = 0.4953)	-0.102 (-0.445–0.24) (*p* = 0.5577)	NG
	Total pts. included	0.031 (-0.073 -0.136) (*p* = 0.5551)	−0.16 (−0.388–0.068) (*p* = 0.1700)	−0.37 (−0.823–0.082) (*p* = 0.1090)
		12 studies included	5 studies included	2 studies included
		597 vs. 2034	88 vs. 528	27 vs. 57
**Glucose**	W/O tPA	0.159 (−0.201–0.52) (*p* = 0.3871)	0.409 (−0.177–0.995) (*p* = 0.1709)	0.429 (−0.251–1.11) (*p* = 0.2163)
	With tPA	0.284 (−0.048–0.617) (*p* = 0.0940)	0.247 (−0.031–0.529) (*p* = 0.0817)	NG
	Total pts. included	**0.189 (0.085–0.293) (*p* = 0.0004)**	**0.315 (0.146–0.483) (*p* = 0.0003)**	**0.418 (0.163–0.673) (*p* = 0.0013)**
		**13 studies included**	**7 studies included**	**4 studies included**
		**708 vs. 3137**	**156 vs. 1769**	**95 vs. 160**
**NIHSS**	W/O tPA	NG	NG	NG
	With tPA	**0.933 (0.358–1.510)**	NG	NG
	Total pts. included	**0.964 (0.477–1.45)**	**0.454 (0.217–0.691)**	**0.438 (0.159–0.718)**
		**6 studies included**	**3 studies included**	**3 studies included**
		**321 vs. 851**	**84 vs. 382**	**84 vs. 124**

NG: Not given; W/O: without. HT: Hemorrhagic Transformation; HI: Hemorrhagic Infarction; PH: Parenchymal Hematoma. HG: Hedges’ G. OR: Odds Ratio. NIHSS: National Institutions of Health Stroke Scale. Numbers marked in bold signify a statistically significant result.

**Table 4:** Follow-up of patients with hemorrhagic transformation.

Variable		HT vs. Non-HT	PH vs. Non-PH	PH vs. HI
**mRS 5–6**	W/O tPA	1.64 (0.93–2.9)	2 (0.3–1.02)	1.55 (0.39–6.12)
		1 study included	1 study included	1 study included
	With tPA	**2.22 (1.7–2.92)**	**6.25 (3.2–12.3)**	**5.53 (2.28–13.40)**
		**2 studies included**	**2 studies included**	**2 studies included**
	Total pts. included	**2.16 (1.7–2.75)**	**5.4 (3.2–9.1)**	**4.25 (2.22–8.14)**
		**4 studies included**	**4 studies included**	**4 studies included**
		**181/646 vs. 207/1192**	**88/165 vs. 300/1673**	**88/165 vs. 93/481**
**mRS 0–1**	W/O tPA	NG	NG	NG
	With tPA	**0.48 (0.25–0.95)**	0.35 (0.01–1.28)	0.5 (0.22–1.15)
		**2 studies included**		
	Total pts. included	**0.45 (0.28–0.71)**	**0.35 (0.15–0.86)**	**0.5 (0.27–0.9)**
		**3 studies included**	**3 studies included**	**3 studies included**
		**92/378 vs. 342/819**	**18/120 vs. 416/1077**	**18/120 vs. 74/258**

NG: Not given; W/O: without. HT: Hemorrhagic Transformation; HI: Hemorrhagic Infarction; PH: Parenchymal Hematoma. OR: Odds Ratio. Numbers marked in bold signify a statistically significant result.

## Data Availability

Questions regarding the data underlying this study should be directed to the corresponding author.

## Supplementary Materials

The following supporting information can be downloaded at https://www.mdpi.com/article/10.3390/jcm11051162/s1.
