# Supplementary material for: Hemorrhagic Transformation in Acute Ischemic Stroke: A Quantitative Systematic Review
Source: J Clin Med. 2022 Feb 22;11(5):1162. doi: 10.3390/jcm11051162 (PMC8910828; doi:10.3390/jcm11051162)
Supplement: Supplementary file 1 [file jcm-11-01162-s001.zip › jcm-1549985-supplementary.pdf]

Table S1. Studies included in the review

Author	Total sample size	Mean age for total sample	Male % for total sample	Imaging modality	Country
Larrue V, et al, 1997 [1]	609, ECASS 1	65	55.4%	CT	14 European countries
Fiorelli M, et al, 1999 [2]	ECASS 1	Not given	Not given	CT	Europe, Australia, New Zealand
Molina CA, et al, 2001 [3]	53	73.7	32%	CT	Spain
Montaner J, et al, 2001 [4]	39	77.6 [4]	50%	CT	Spain
Larrue V, et al, 2001 [5]	ECASS 2	Not given	Not given	CT	Europe, Australia, New Zealand
Hermier M, et al, 2003 [6]	58	63	43.1%	MRI	France
Montaner J, et al, [7]	41	70+/-10.6	39%	CT	Spain
Montaner J, et al, 2003 [8]	61	71+/-9.3	44.3%	CT	Spain
Celik Y, et al, 2004 [9]	86 Consecutive MCA pts, no antithrombotic tx	68.1	47.7%	CT	Turkey
Castellanos M, et al, 2004 [10]	87	67	69.8%	CT	Spain
Ribo M, et al, 2004 [11]	77	70	60%	CT	Spain
Rodriguez-Yanez M, et al, 2006 [12]	200	72.5	51.5%	CT	Spain
IMS study group, 2006 [13]	80	Not given	Not given	CT	USA
Dzialowski I, et al, 2007 [14]	954	67.1	55%	CT	Canada
Millan M, et al, 2007 [15]	134	67	65.7	CT	France, Spain
Thomalla G, et al, 2007 [16]	152	64	61.2%	CT/MRI	Germany
Paciaroni M, et al, 2008 [17]	1125 consecutive AIS pts	76	55.9%	CT	Italy
Kimura K, et al, 2008 [18]	51	73	43%	MRI	Japan
Hjort N, et al, 2008 [19]	33	68	Not given	MRI	Denmark
Mendioroz M, et al, 2009 [20]	119	71.8	59%	CT	Spain
Aviv RI, et al, 2009 [21]	41	70.3+15.4	63%	MRI	Canada
Butcher K, et al, 2010 [22]	97, EPIPHET RCT	71.2+14	55%	MRI	Canada, Australia, New Zealand
Campbell BCV, et al, 2010 [23]	91, EPIPHET RCT	71.4	53.8%	MRI	Australia, UK, New Zealand, Belgium

Kim JH, et al, 2010 [24]	184 consecutive MCA AIS pts	65.7	53.8%	MRI	USA<br>S Korea
Prodan CI, et al, 2010 [25]	115	64.6	71%	CT/MRI	USA
Hernandez-Guillamon M, et al, 2010 [26]	141	Not given	Not given	CT	Spain
Sobrino T, et al, 2010 [27]	79	66.7	54.4%	CT	Spain
Ko Y, et al, 2010 [28]	786 consecutive AIS pts	70	60.6%	CT/MRI	S Korea
Choi KH, et al, 2012 [29]	752 consecutive first AIS pts	65.9	59.9%	MRI	Korea
Leira R, et al, 2012 [30]	161	70.6	Not given	MRI	Spain
del Rio-Espínola A, et al, 2012 [31]	885	Not given	75.5%	CT	Spain
El-Khawas HM, et al, 2012 [32]	324 consecutive AIS pts.	60.5	Not given	MRI	Egypt
Costello CA, et al, 2012 [33]	206	71	68.4%	CT	Australia
Takahashi W, et al, 2013 [34]	187	74	40.1%	CT/MRI	Japan
Jain AR, et al, 2013 [35]	83 consecutive AIS pts	72	55.4%	CT/MRI	USA
Kufner A, et al, 2013 [36]	109	71.3+-12.5	50%	MRI	Germany
Lee JG, et al, [37]	770 consecutive AIS pts.	70.3	56.1%	MRI	Korea
Yassi N, et al, 2013 [38]	132	74	Not given	CT/MRI	Australia
Pereira VM, et al, 2013 [39]	202, STAR clinical trial	72	40%	CT/MRI	Europe, Canada, Australia
Rodriguez-Gonzalez R, et al, 2013 [40]	129	66.5	56.7%	CT	Spain
Jickling GC, et al, 2013 [41]	44, CLEAR trial	68.7	63.6%	CT	USA
Cortijo E, et al, 2014 [42]	69	71.8	52%	CT	Spain
Hong JM, et al, 2014 [43]	75	66.2	59%	CT	S Korea
Llombart V, et al, 2014 [44]	186	71.5	53.8%	CT	Spain
Öcek L, et al 2015 [45]	171	71.2	55%	CT/MRI	Turkey
d'Esterre C, et al, 2015 [46]	55	67.3	41.8%	CT	Canada, Italy
Renú A, et al, 2015 [47]	132 endovascular therapy	67	47.7%	CT/MRI	Spain
Liu C, et al, 2015, [48]	87	67.3	56.3%	MRI	China
Guo Y, et al, 2015 [49]	362	67.7	63.2%	CT/MRI	China

Jia W, et al, 2015 [50]	904	63.6	38.3%	CT	China
Maeshima S, et al, 2016 [51]	165	Not given	38.1%	CT	Japan
Shi ZS, et al, 2016 [52]	206 endovascular therapy	66.8	42.2%	CT/MRI	Korea
Bivard A, et al, 2016 [53]	229	68	Not given	MRI	Australia and China
Cappellari M, et al, 2016 [54]	147	79	38%	CT	Italy
Purrucker J, et al, 2016 [55]	28	74	54%	CT/MRI	Germany
Gioia L, et al, 2016 [56]	66	71	67%	MRI	Canada
Heo J, et al, 2016 [57]	289 EUREKA RCT	64.5	59.9%	MRI	Korea
Liu J, et al, 2016 [58]	130	68.15	43.1%	MRI	China
Ho BL, et al, 2016 [59]	241 TTT-AIS Study	65.8	60.2%	CT	Taiwan
Guo Z, et al, 2016 [60]	189	65	65%	CT	China
Bas DF, et al, 2016 [61]	180	63.1	62%	CT	Turkey
Chen X, et al, 2017 [62]	316	66.3	62.3%	CT	China
Wei CC, et al, 2017 [63]	251	68.5	39.4%	MRI	China
Castro P, et al, 2017 [64]	46	73	54%	CT	Portugal
Purrucker JC, et al, 2017 [65]	231	77.4	53.2%	CT/MRI	Germany

Table S2. Studies Included in Individual Analyses.

**Study Characteristics**	**Number**
Number of studies analyzing HT with CT	**34**
Number of studies analyzing HT with MRI	18
Number of studies analyzing HT with a combination of CT and MRI	13
Number of studies published before 2000	3
Number of studies published after 2000	62
Number of studies including data from East Asia	19
Number of studies including data from Europe	35
Number of studies including data from North America	11
Number of studies including data from the Middle East (Egypt)	1

Table S3. Baseline Patient Characteristics

Variable		HT vs non-HT	PH vs non-PH	PH vs HI
**Baseline patient characteristics**				
**Age**<br>**HG**	W/O tPA	0.1 (-0.124–0.325)<br>3 studies [12, 25, 30]	0.121 (-0.516–0.758)<br>1 study [30]	0.115 (-0.56–0.791)<br>1 study [30]
	With tPA	0.3 (-0.249–0.849)<br>4 studies [10, 18, 26, 41]	**0.271** (0.037–0.505)<br>5 studies [20, 26, 27, 40, 59]<br>102 vs 552	NG
	Total pts. included	**0.125** (0.048–0.202)<br>15 studies [1, 9, 10, 12, 18, 22, 24-26, 30, 32, 37, 41, 52, 62]<br>968 vs 2512	**0.215** (0.087–0.344)<br>10 studies [1, 20, 22, 24, 26, 27, 30, 40, 52, 60]<br>273 vs 1729	**0.267** (0.084–0.45)<br>5 studies [1, 22, 24, 30, 52]<br>161 vs 386
**Male**<br>**OR**	W/O tPA	1.71 (0.55–5.33)<br>3 studies [3, 12, 25]	5.07 (0.48–54.02)<br>1 study [8]	4.16 (0.33–50)<br>1 study [8]
	With tPA	0.89 (0.7–1.14)<br>5 studies [10, 12, 14, 25, 26]	1.37 (0.78–2.40)<br>7 studies [9, 13, 14, 26, 27, 40, 60]	1.08 (1.92–0.61)<br>2 studies [14, 26]
	Total pts. included	1.07 (0.88–1.31)<br>18 studies [1, 3, 10, 12, 14, 17, 18, 23-26, 29, 37, 41, 52, 56, 62, 64]<br>744/1282 vs 2568/4527	**1.50 (1.07–2.11)**<br>14 studies [1, 7, 9, 13, 14, 17, 23, 24, 26, 27, 29, 40, 52, 60]<br>281/431 vs 2395/4263	**1.43 (1–2)**<br>9 studies [1, 7, 14, 17, 23, 24, 26, 29, 52]<br>204/326 vs 352/640
**Chronic hypertension**<br>**OR**	W/O tPA	1.67 (0.52–5.32)<br>4 studies [3, 12, 30, 45]	1.74 (0.54–5.54)<br>2 studies [30, 45]	2.69 (0.85–8.53)<br>3 studies [7, 30, 45]
	With tPA	1.74 (0.85–3.55)<br>2 studies included [14, 26]	**1.51 (1.10–2.07)**<br>6 studies [14, 20, 26, 27, 40, 60]<br>124/194 vs 745/1414	1.54 (0.96–2.48)<br>2 studies [14, 26]<br>64/108 vs 97/199
	Total pts. included	1.21 (0.86–1.71)<br>11 studies [1, 3, 12, 14, 17, 24, 26, 29, 30, 38, 45]<br>562/996 vs 2170/3485	**1.29 (1.04–1.61)**<br>12 studies [1, 14, 17, 20, 24, 26, 27, 29, 38, 40, 45, 60]<br>248/392 vs 2665/4350	1.31 (0.87–1.96)<br>8 studies [1, 14, 17, 24, 26, 29, 30, 38]<br>192/310 vs 340/649
**Previous Diabetes**<br>**OR**	W/O tPA	1.41 (0.66–3.03)<br>5 studies [3, 12, 25, 30, 45]	NG	2.4 (0.96–6.02)<br>3 studies [7, 30, 45]
	With tPA	0.97 (0.69–1.38)<br>4 studies [14, 18, 26, 41]	NG	1 (0.1–9.91)<br>2 studies [14, 26]
	Total pts. included	1.23 (0.97–1.56)<br>21 studies [1, 3, 9, 12, 14, 17, 18, 22-26, 28-30, 37, 38, 41, 45, 62, 63]<br>339/1466 vs 1318/5571	NG	**1.66 (1.05–2.61)**<br>11 studies [1, 7, 14, 17, 23, 24, 26, 29, 30, 38, 45]<br>83/323 vs 117/680
**Hyperlipidemia**<br>**OR**	W/O tPA	**0.53 (0.31–0.93)**<br>3 studies [3, 30, 45]	0.81 (0.29–2.26)<br>2 studies [30, 45]	1.14 (0.37–3.46)<br>3 studies [7, 30, 45]

	With tPA	0.52 (0.25–1.06)<br>2 studies [26, 41]	1.25 (0.61–2.58)<br>5 studies [20, 26, 27, 40, 60]	0.82 (0.18–3.73)<br>1 study [26]
	Total pts. included	0.8 (0.62–1.03)<br>10 studies [3, 26, 28, 30, 37, 38, 41, 45, 62, 64]<br>101/452 vs 529/2167	1.02 (0.57–1.84)<br>8 studies [20, 26, 27, 30, 38, 40, 45, 60]<br>56/138 vs 338/980	0.76 (0.33–1.75)<br>5 studies [7, 26, 30, 38, 45]<br>9/56 vs 29/136
**Alcohol Abuse**<br>**OR**	W/O tPA	1.47 (0.55–3.9)<br>1 study	1.97 (0.39–10.05)<br>1 study	1.62 (0.28–9.56)<br>1 study
	With tPA	NG	NG	NG
	Total pts. included	1.32 (0.92–1.88)<br>4 studies [17, 29, 30, 63]<br>56/342 vs 228/1947	1.02 (0.5–2.08)<br>3 studies [17, 29, 30]<br>10/78 vs 225/1960	1.15 (0.49–2.72)<br>3 studies [17, 29, 30]<br>10/78 vs 20/165
**Prev. anticoagulation**<br>**OR**	W/O tPA	NG	NG	NG
	With tPA	NG	NG	NG
	Total pts. included	**2.47 (1.64–3.72)**<br>4 studies [17, 32, 37, 63]<br>40/378 vs 86/2092	**2.9 (1–8.55)**<br>1 study [17]<br>4/36 vs 46/1089	1.81 (0.42–7.74)<br>1 study [17]<br>4/36 vs 4/62
**Prev. Antiplatelets**<br>**OR**	**W/O tPA**	0.64 (0.18–2.32)<br>1 study [25]	NG	NG
	**With tPA**	1.18 (0.84–1.66)<br>1 study [14]	**3.15 (1.39–7.17)**<br>4 studies [14, 27, 40, 60]<br>61/165 vs 237/1186	1.43 (0.8–2.57)<br>1 study [14]
	**Total pts. included**	1.01 (0.83–1.23)<br>8 studies [1, 17, 25, 32, 37, 55, 62, 63]<br>232/937 vs 1080/3527	**2.25 (1.26–4.02)**<br>6 studies [1, 14, 17, 27, 40, 60]<br>85/263 vs 630/2822	1.38 (0.66–2.87)<br>3 studies [1, 14, 17]<br>50/190 vs 78/414
**Prev. Statins**<br>**OR**	**W/O tPA**	0.36 (0.45–1.29) (25)	NG	NG
	**With tPA**	NG	**3.58 (1.41–9.05)**<br>2 studies [27, 40]	NG
	**Total pts. included**	0.76 (0.45–1.29)<br>6 studies [17, 24, 25, 37, 57, 63]<br>54/440 vs 366/2294	2.15 (0.98–4.76)<br>4 studies[17, 24, 27, 40]<br>31/113 vs 146/1404	1.27 (0.2–8.15)<br>2 studies [17, 24]<br>10/68 vs 11/103
**Atrial Fibrillation**<br>**OR**	**W/O tPA**	**4 (1.00–15.92)**	5.1 (0.69–37.86)	**4.27 (1.13–16.23)**
	**With tPA**	1.98 (0.55–7.14)	1.26 (0.83–1.9)	0.75 (0.41–1.38)
	**Total pts. included**	**2.85 (1.86–4.38)**<br>15 studies [1, 12, 14, 17, 18, 22, 23, 29, 30, 32, 38, 41, 45, 62, 64]<br>381/1039 vs 795/3943	**2.25 (1.47–3.44)**<br>12 studies [1, 14, 17, 22, 23, 27, 29, 30, 38, 45, 60]<br>146/359 vs 942/4156	**2.09 (1.26–3.48)**<br>9 studies [1, 14, 17, 22, 23, 29, 30, 38, 45]<br>118/286 vs 186/627
**LDL level**<br>**HG**	**W/O tPA**	**-0.657 (-0.288– -1.03)**<br>1 study [45]	-0.089 (-0.673–0.496)<br>1 study [45]	0.234 (-0.442–0.909)<br>1 study [45]
	**With tPA**	NG	NG	NG
	Total pts. included	**-0.304 (-0.124– -0.483)**	-0.075 (-0.304–0.155)	0.018 (-0.259–0.296)

		5 studies [17, 24, 32, 45, 63]<br>**356 vs 1699**	3 studies [17, 24, 45]<br>80 vs 1400	3 studies [17, 24, 45]<br>80 vs 128

Table S4. Patient data upon admission

Variable		HT vs non-HT	PH vs non-PH	PH vs HI
**Systolic BP**	W/O tPA	-0.101 (-0.407–0.202)	-0.445 (-1.032–0.14)	**-0.759 (-0.063– -1.454)<br>1 study [45]<br>12 vs 25**
	With tPA	0.05 (-0.188–0.285)	**0.34 (0.11–0.569)<br>4 studies [26, 27, 40, 60]<br>89 vs 448**	NG
	Total pts.	0.043 (-0.056–0.141)<br>12 studies [3, 9, 10, 18, 22, 26, 28, 37, 41, 45, 63, 64]<br>547 vs 2035	**0.217 (-0.02–0.454)<br>6 studies [22, 26, 27, 40, 45, 60]<br>116 vs 689**	-0.227 (-1.24–0.786)<br>2 studies [22, 45]<br>27 vs 57
**Diastolic BP**	W/O tPA	-0.213 (-0.519–0.094)	-0.353 (-0.94–0.232)	-0.483 (-1.166–0.2)
	With tPA	0.08 (-0. 15–0.312)	-0.102 (-0.445–0.24)	NG
	Total pts.	0.031 (-0.073–0.136)<br>12 studies [3, 9, 10, 18, 22, 26, 28, 37, 41, 45, 63, 64]<br>597 vs 2034	-0.16 (-0.388–0.068)<br>5 studies [22, 26, 27, 40, 45]<br>88 vs 528	-0.37 (-0.823–0.082)<br>2 studies [22, 45]<br>27 vs 57
**Glucose**	W/O tPA	0.159 (-0.201–0.52)	0.409 (-0.177–0.995)	0.429 (-0.251–1.11)
	With tPA	0.284 (-0.048–0.617)	0.247 (-0.031–0.529)	NG
	Total pts.	**0.189 (0.085–0.293)<br>13 studies [3, 9, 10, 17, 18, 22, 24, 26, 28, 37, 41, 45, 63]<br>708 vs 3137**	**0.315 (0.146–0.483)<br>7 studies [17, 22, 24, 26, 27, 40, 45]<br>156 vs 1769**	**0.418 (0.163–0.673)<br>4 studies [17, 22, 24, 45]<br>95 vs 160**
**NIHSS**	W/O tPA	NG	NG	NG
	With tPA	**0.933 (0.358–1.510)**	NG	NG
	Total pts. included	**0.964 (0.477–1.45)<br>6 studies [18, 23, 24, 32, 52, 62]<br>321 vs 851**	**0.454 (0.217–0.691)<br>3 studies [23, 24, 52]<br>84 vs 382**	**0.438 (0.159–0.718)<br>3 studies [23, 24, 52]<br>84 vs 124**

Table S5. Prognostic follow-up of patients with hemorrhagic transformation

**Variable**		**HT vs non-HT**	**PH vs non-PH**	**PH vs HI**
**mRS 5-6**	W/O tPA	1.64 (0.93–2.9)<br>1 study [5]	2 (0.3–1.02)<br>1 study [5]	1.55 (0.39–6.12)<br>1 study [5]
	With tPA	**2.22 (1.7–2.92)**<br>**2 studies [5, 14]**	**6.25 (3.2–12.3)**<br>**2 studies [5, 14]**	**5.53 (2.28–13.40)**<br>**2 studies [5, 14]**
	All patients	**2.16 (1.7–2.75)**<br>**4 studies [1, 5, 14, 23]**<br>**181/646 vs 207/1192**	**5.4 (3.2–9.1)**<br>**4 studies [1, 5, 14, 23]**<br>**88/165 vs 300/1673**	**4.25 (2.22–8.14)**<br>**4 studies [1, 5, 14, 23]**<br>**88/165 vs 93/481**
**mRS 0-1**	W/O tPA	NG	NG	NG
	With tPA	**0.48 (0.25–0.95)**<br>**2 studies [14, 16]**	0.35 (0.10–1.28)<br>2 studies [14, 16]	0.5 (0.22–1.15)<br>2 studies [14, 16]
	All patients	**0.45 (0.28–0.71)**<br>**3 studies [14, 16, 23]**<br>**92/378 vs 342/819**	**0.35 (0.15–0.86)**<br>**3 studies [14, 16, 23]**<br>**18/120 vs 416/1077**	**0.5 (0.27–0.9)**<br>**3 studies [14, 16, 23]**<br>**18/120 vs 74/258**

1. Larrue, V.; von Kummer, R.; del Zoppo, G.; Bluhmki, E., Hemorrhagic transformation in acute ischemic stroke. Potential contributing factors in the European Cooperative Acute Stroke Study. *Stroke* **1997,** *28* (5), 957-960.

2. Fiorelli, M.; Bastianello, S.; von Kummer, R.; del Zoppo, G. J.; Larrue, V.; Lesaffre, E.; Ringleb, A. P.; Lorenzano, S.; Manelfe, C.; Bozzao, L., Hemorrhagic transformation within 36 hours of a cerebral infarct: relationships with early clinical deterioration and 3-month outcome in the European Cooperative Acute Stroke Study I (ECASS I) cohort. *Stroke* **1999,** *30* (11), 2280-2284.

3. Molina, C. A.; Montaner, J.; Abilleira, S.; Ibarra, B.; Romero, F.; Arenillas, J. F.; Alvarez-Sabín, J., Timing of spontaneous recanalization and risk of hemorrhagic transformation in acute cardioembolic stroke. *Stroke* **2001,** *32* (5), 1079-1084.

4. Montaner, J.; Alvarez-Sabín, J.; Molina, C. A.; Anglés, A.; Abilleira, S.; Arenillas, J.; Monasterio, J., Matrix metalloproteinase expression is related to hemorrhagic transformation after cardioembolic stroke. *Stroke* **2001,** *32* (12), 2762-2767.

5. Larrue, V.; von Kummer, R. R.; Müller, A.; Bluhmki, E., Risk factors for severe hemorrhagic transformation in ischemic stroke patients treated with recombinant tissue plasminogen activator: a secondary analysis of the European-Australasian Acute Stroke Study (ECASS II). *Stroke* **2001,** *32* (2), 438-441.

6. Hermier, M.; Nighoghossian, N.; Derex, L.; Adeleine, P.; Wiart, M.; Berthezène, Y.; Cotton, F.; Pialat, J. B.; Dardel, P.; Honnorat, J.; Trouillas, P.; Froment, J. C., Hypointense transcerebral veins at T2*-weighted MRI: a marker of hemorrhagic transformation risk in patients treated with intravenous tissue plasminogen activator. *J Cereb Blood Flow Metab* **2003,** *23* (11), 1362-1370.

7. Montaner, J.; Molina, C. A.; Monasterio, J.; Abilleira, S.; Arenillas, J. F.; Ribó, M.; Quintana, M.; Alvarez-Sabín, J., Matrix metalloproteinase-9 pretreatment level predicts intracranial hemorrhagic complications after thrombolysis in human stroke. *Circulation* **2003,** *107* (4), 598-603.

8. Montaner, J.; Fernández-Cadenas, I.; Molina, C. A.; Monasterio, J.; Arenillas, J. F.; Ribó, M.; Quintana, M.; Chacón, P.; Andreu, A. L.; Alvarez-Sabín, J., Safety profile of tissue plasminogen activator treatment among stroke patients carrying a common polymorphism (C-1562T) in the promoter region of the matrix metalloproteinase-9 gene. *Stroke* **2003,** *34* (12), 2851-2855.

9. Celik, Y.; Utku, U.; Asil, T.; Balci, K., Factors affecting haemorrhagic transformation in middle cerebral artery infarctions. *J Clin Neurosci* **2004,** *11* (6), 656-658.

10. Castellanos, M.; Leira, R.; Serena, J.; Blanco, M.; Pedraza, S.; Castillo, J.; Dávalos, A., Plasma cellular-fibronectin concentration predicts hemorrhagic transformation after thrombolytic therapy in acute ischemic stroke. *Stroke* **2004,** *35* (7), 1671-1676.

11. Ribo, M.; Montaner, J.; Molina, C. A.; Arenillas, J. F.; Santamarina, E.; Quintana, M.; Alvarez-Sabín, J., Admission fibrinolytic profile is associated with symptomatic hemorrhagic transformation in stroke patients treated with tissue plasminogen activator. *Stroke* **2004,** *35* (9), 2123-2127.

12. Rodríguez-Yáñez, M.; Castellanos, M.; Blanco, M.; Millán, M.; Nombela, F.; Sobrino, T.; Lizasoain, I.; Leira, R.; Serena, J.; Dávalos, A.; Castillo, J., Micro- and macroalbuminuria predict hemorrhagic transformation in acute ischemic stroke. *Neurology* **2006,** *67* (7), 1172-1177.

13. Hemorrhage in the interventional management of stroke study. *Stroke* **2006,** *37* (3), 847-851.

14. Dzialowski, I.; Pexman, J. H.; Barber, P. A.; Demchuk, A. M.; Buchan, A. M.; Hill, M. D., Asymptomatic hemorrhage after thrombolysis may not be benign: prognosis by hemorrhage type in the Canadian alteplase for stroke effectiveness study registry. *Stroke* **2007,** *38* (1), 75-79.

15. Millan, M.; Sobrino, T.; Castellanos, M.; Nombela, F.; Arenillas, J. F.; Riva, E.; Cristobo, I.; García, M. M.; Vivancos, J.; Serena, J.; Moro, M. A.; Castillo, J.; Dávalos, A., Increased body iron stores are associated with poor outcome after thrombolytic treatment in acute stroke. *Stroke* **2007,** *38* (1), 90-95.

16. Thomalla, G.; Sobesky, J.; Köhrmann, M.; Fiebach, J. B.; Fiehler, J.; Zaro Weber, O.; Kruetzelmann, A.; Kucinski, T.; Rosenkranz, M.; Röther, J.; Schellinger, P. D., Two tales: hemorrhagic transformation but not parenchymal hemorrhage after thrombolysis is related to severity and duration of ischemia: MRI study of acute stroke patients treated with intravenous tissue plasminogen activator within 6 hours. *Stroke* **2007,** *38* (2), 313-318.

17. Paciaroni, M.; Agnelli, G.; Corea, F.; Ageno, W.; Alberti, A.; Lanari, A.; Caso, V.; Micheli, S.; Bertolani, L.; Venti, M.; Palmerini, F.; Biagini, S.; Comi, G.; Previdi, P.; Silvestrelli, G., Early hemorrhagic transformation of brain infarction: rate, predictive factors, and influence on clinical outcome: results of a prospective multicenter study. *Stroke* **2008,** *39* (8), 2249-2256.

18. Kimura, K.; Iguchi, Y.; Shibazaki, K.; Aoki, J.; Terasawa, Y., Hemorrhagic transformation of ischemic brain tissue after t-PA thrombolysis as detected by MRI may be asymptomatic, but impair neurological recovery. *J Neurol Sci* **2008,** *272* (1-2), 136-142.

19. Hjort, N.; Wu, O.; Ashkanian, M.; Sølling, C.; Mouridsen, K.; Christensen, S.; Gyldensted, C.; Andersen, G.; Østergaard, L., MRI detection of early blood-brain barrier disruption: parenchymal enhancement predicts focal hemorrhagic transformation after thrombolysis. *Stroke* **2008,** *39* (3), 1025-1028.

20. Mendioroz, M.; Fernández-Cadenas, I.; Alvarez-Sabín, J.; Rosell, A.; Quiroga, D.; Cuadrado, E.; Delgado, P.; Rubiera, M.; Ribó, M.; Molina, C.; Montaner, J., Endogenous activated protein C predicts hemorrhagic transformation and mortality after tissue plasminogen activator treatment in stroke patients. *Cerebrovasc Dis* **2009,** *28* (2), 143-150.

21. Aviv, R. I.; d'Esterre, C. D.; Murphy, B. D.; Hopyan, J. J.; Buck, B.; Mallia, G.; Li, V.; Zhang, L.; Symons, S. P.; Lee, T. Y., Hemorrhagic transformation of ischemic stroke: prediction with CT perfusion. *Radiology* **2009,** *250* (3), 867-877.

22. Butcher, K.; Christensen, S.; Parsons, M.; De Silva, D. A.; Ebinger, M.; Levi, C.; Jeerakathil, T.; Campbell, B. C.; Barber, P. A.; Bladin, C.; Fink, J.; Tress, B.; Donnan, G. A.; Davis, S. M., Postthrombolysis blood pressure elevation is associated with hemorrhagic transformation. *Stroke* **2010,** *41* (1), 72-77.

23. Campbell, B. C.; Christensen, S.; Butcher, K. S.; Gordon, I.; Parsons, M. W.; Desmond, P. M.; Barber, P. A.; Levi, C. R.; Bladin, C. F.; De Silva, D. A.; Donnan, G. A.; Davis, S. M., Regional very low cerebral blood volume predicts hemorrhagic transformation better than diffusion-weighted imaging volume and thresholded apparent diffusion coefficient in acute ischemic stroke. *Stroke* **2010,** *41* (1), 82-88.

24. Kim, J. H.; Bang, O. Y.; Liebeskind, D. S.; Ovbiagele, B.; Kim, G. M.; Chung, C. S.; Lee, K. H.; Saver, J. L., Impact of baseline tissue status (diffusion-weighted imaging lesion) versus perfusion status (severity of hypoperfusion) on hemorrhagic transformation. *Stroke* **2010,** *41* (3), e135-142.

25. Prodan, C. I.; Stoner, J. A.; Cowan, L. D.; Dale, G. L., Lower coated-platelet levels are associated with early hemorrhagic transformation in patients with non-lacunar brain infarction. *J Thromb Haemost* **2010,** *8* (6), 1185-1190.

26. Hernandez-Guillamon, M.; Garcia-Bonilla, L.; Solé, M.; Sosti, V.; Parés, M.; Campos, M.; Ortega-Aznar, A.; Domínguez, C.; Rubiera, M.; Ribó, M.; Quintana, M.; Molina, C. A.; Alvarez-Sabín, J.; Rosell, A.; Unzeta, M.; Montaner, J., Plasma VAP-1/SSAO activity predicts intracranial hemorrhages and adverse neurological outcome after tissue plasminogen activator treatment in stroke. *Stroke* **2010,** *41* (7), 1528-1535.

27. Sobrino, T.; Millán, M.; Castellanos, M.; Blanco, M.; Brea, D.; Dorado, L.; Rodríguez-González, R.; Rodríguez-Yáñez, M.; Serena, J.; Leira, R.; Dávalos, A.; Castillo, J., Association of growth factors with arterial recanalization and clinical outcome in patients with ischemic stroke treated with tPA. *J Thromb Haemost* **2010,** *8* (7), 1567-1574.

28. Ko, Y.; Park, J. H.; Yang, M. H.; Ko, S. B.; Han, M. K.; Oh, C. W.; Lee, J.; Lee, J.; Bae, H. J., The significance of blood pressure variability for the development of hemorrhagic transformation in acute ischemic stroke. *Stroke* **2010,** *41* (11), 2512-2518.

29. Choi, K. H.; Park, M. S.; Kim, J. T.; Nam, T. S.; Choi, S. M.; Kim, B. C.; Kim, M. K.; Cho, K. H., The serum ferritin level is an important predictor of hemorrhagic transformation in acute ischaemic stroke. *Eur J Neurol* **2012,** *19* (4), 570-577.

30. Leira, R.; Sobrino, T.; Blanco, M.; Campos, F.; Rodríguez-Yáñez, M.; Castellanos, M.; Moldes, O.; Millán, M.; Dávalos, A.; Castillo, J., A higher body temperature is associated with haemorrhagic transformation in patients with acute stroke untreated with recombinant tissue-type plasminogen activator (rtPA). *Clin Sci (Lond)* **2012,** *122* (3), 113-119.

31. del Río-Espínola, A.; Fernández-Cadenas, I.; Giralt, D.; Quiroga, A.; Gutiérrez-Agulló, M.; Quintana, M.; Fernández-Álvarez, P.; Domingues-Montanari, S.; Mendióroz, M.; Delgado, P.; Turck, N.; Ruíz, A.; Ribó, M.; Castellanos, M.; Obach, V.; Martínez, S.; Freijo, M. M.; Jiménez-Conde, J.; Cuadrado-Godia, E.; Roquer, J.; Chacón, P.; Martí-Fábregas, J.; Sánchez, J. C.; Montaner, J., A predictive clinical-genetic model of tissue plasminogen activator response in acute ischemic stroke. *Ann Neurol* **2012,** *72* (5), 716-729.

32. El-Khawas, H. M.; El-Rakawy, M. H.; Zakaria, M. F.; Tantawy, W. H.; Raafat, M. A.; Fouad, M. M., Predictive factors of hemorrhagic transformation in acute ischemic stroke. *Egyptian Journal of Neurology, Psychiatry & Neurosurgery* **2012,** *49* (3).

33. Costello, C. A.; Campbell, B. C.; Perez de la Ossa, N.; Zheng, T. H.; Sherwin, J. C.; Weir, L.; Hand, P.; Yan, B.; Desmond, P. M.; Davis, S. M., Age over 80 years is not associated with increased hemorrhagic transformation after stroke thrombolysis. *J Clin Neurosci* **2012,** *19* (3), 360-363.

34. Takahashi, W.; Moriya, Y.; Mizuma, A.; Uesugi, T.; Ohnuki, Y.; Takizawa, S., Cerebral microbleeds on T2*-weighted images and hemorrhagic transformation after antithrombotic therapies for ischemic stroke. *J Stroke Cerebrovasc Dis* **2013,** *22* (8), e528-532.

35. Jain, A. R.; Jain, M.; Kanthala, A. R.; Damania, D.; Stead, L. G.; Wang, H. Z.; Jahromi, B. S., Association of CT perfusion parameters with hemorrhagic transformation in acute ischemic stroke. *AJNR Am J Neuroradiol* **2013,** *34* (10), 1895-1900.

36. Kufner, A.; Galinovic, I.; Brunecker, P.; Cheng, B.; Thomalla, G.; Gerloff, C.; Campbell, B. C.; Nolte, C. H.; Endres, M.; Fiebach, J. B.; Ebinger, M., Early infarct FLAIR hyperintensity is associated with increased hemorrhagic transformation after thrombolysis. *Eur J Neurol* **2013,** *20* (2), 281-285.

37. Lee, J. G.; Lee, K. B.; Jang, I. M.; Roh, H.; Ahn, M. Y.; Woo, H. Y.; Hwang, H. W., Low glomerular filtration rate increases hemorrhagic transformation in acute ischemic stroke. *Cerebrovasc Dis* **2013,** *35* (1), 53-59.

38. Yassi, N.; Parsons, M. W.; Christensen, S.; Sharma, G.; Bivard, A.; Donnan, G. A.; Levi, C. R.; Desmond, P. M.; Davis, S. M.; Campbell, B. C., Prediction of poststroke hemorrhagic transformation using computed tomography perfusion. *Stroke* **2013,** *44* (11), 3039-3043.

39. Pereira, V. M.; Gralla, J.; Davalos, A.; Bonafé, A.; Castaño, C.; Chapot, R.; Liebeskind, D. S.; Nogueira, R. G.; Arnold, M.; Sztajzel, R.; Liebig, T.; Goyal, M.; Besselmann, M.; Moreno, A.; Moreno, A.; Schroth, G., Prospective, multicenter, single-arm study of mechanical thrombectomy using Solitaire Flow Restoration in acute ischemic stroke. *Stroke* **2013,** *44* (10), 2802-2807.

40. Rodríguez-González, R.; Blanco, M.; Rodríguez-Yáñez, M.; Moldes, O.; Castillo, J.; Sobrino, T., Platelet derived growth factor-CC isoform is associated with hemorrhagic transformation in ischemic stroke patients treated with tissue plasminogen activator. *Atherosclerosis* **2013,** *226* (1), 165-171.

41. Jickling, G. C.; Ander, B. P.; Stamova, B.; Zhan, X.; Liu, D.; Rothstein, L.; Verro, P.; Khoury, J.; Jauch, E. C.; Pancioli, A. M.; Broderick, J. P.; Sharp, F. R., RNA in blood is altered prior to hemorrhagic transformation in ischemic stroke. *Ann Neurol* **2013,** *74* (2), 232-240.

42. Cortijo, E.; García-Bermejo, P.; Calleja, A. I.; Pérez-Fernández, S.; Gómez, R.; del Monte, J. M.; Reyes, J.; Arenillas, J. F., Intravenous thrombolysis in ischemic stroke with unknown onset using CT perfusion. *Acta Neurol Scand* **2014,** *129* (3), 178-183.

43. Hong, J. M.; Lee, J. S.; Song, H. J.; Jeong, H. S.; Choi, H. A.; Lee, K., Therapeutic hypothermia after recanalization in patients with acute ischemic stroke. *Stroke* **2014,** *45* (1), 134-140.

44. Llombart, V.; Dominguez, C.; Bustamante, A.; Rodriguez-Sureda, V.; Martín-Gallán, P.; Vilches, A.; García-Berrocoso, T.; Penalba, A.; Hernández-Guillamon, M.; Rubiera, M.; Ribó, M.; Eschenfelder, C.; Giralt, D.; Molina, C. A.; Alvarez-Sabín, J.; Rosell, A.; Montaner, J.,

Fluorescent molecular peroxidation products: a prognostic biomarker of early neurologic deterioration after thrombolysis. *Stroke* **2014,** *45* (2), 432-437.

45. Öcek, L.; Güner, D.; Uludağ İ, F.; Tiftikçioğlu, B.; Zorlu, Y., Risk factors for hemorrhagic transformation in patients with acute middle cerebral artery infarction. *Noro Psikiyatr Ars* **2015,** *52* (4), 342-345.

46. d'Esterre, C. D.; Roversi, G.; Padroni, M.; Bernardoni, A.; Tamborino, C.; De Vito, A.; Azzini, C.; Marcello, O.; Saletti, A.; Ceruti, S.; Lee, T. Y.; Fainardi, E., CT perfusion cerebral blood volume does not always predict infarct core in acute ischemic stroke. *Neurol Sci* **2015,** *36* (10), 1777-1783.

47. Renú, A.; Amaro, S.; Laredo, C.; Román, L. S.; Llull, L.; Lopez, A.; Urra, X.; Blasco, J.; Oleaga, L.; Chamorro, Á., Relevance of blood-brain barrier disruption after endovascular treatment of ischemic stroke: dual-energy computed tomographic study. *Stroke* **2015,** *46* (3), 673-679.

48. Liu, C.; Dong, Z.; Xu, L.; Khursheed, A.; Dong, L.; Liu, Z.; Yang, J.; Liu, J., MR image features predicting hemorrhagic transformation in acute cerebral infarction: a multimodal study. *Neuroradiology* **2015,** *57* (11), 1145-1152.

49. Guo, Y.; Yan, S.; Zhang, S.; Zhang, X.; Chen, Q.; Liu, K.; Liebeskind, D. S.; Lou, M., Lower serum calcium level is associated with hemorrhagic transformation after thrombolysis. *Stroke* **2015,** *46* (5), 1359-1361.

50. Jia, W.; Liao, X.; Pan, Y.; Wang, Y.; Cui, T.; Zhou, L.; Wang, Y., Thrombolytic-related asymptomatic hemorrhagic transformation does not deteriorate clinical outcome: data from TIMS in China. *PLoS One* **2015,** *10* (11), e0142381.

51. Maeshima, S.; Okamoto, S.; Okazaki, H.; Mizuno, S.; Asano, N.; Tsunoda, T.; Maeda, H.; Masaki, M.; Sonoda, S., Hemorrhagic transformation in patients with cerebral infarction referred to a rehabilitation hospital. *Interv Neurol* **2016,** *4* (3-4), 69-74.

52. Shi, Z. S.; Duckwiler, G. R.; Jahan, R.; Tateshima, S.; Gonzalez, N. R.; Szeder, V.; Saver, J. L.; Kim, D.; Ali, L. K.; Starkman, S.; Vespa, P. M.; Salamon, N.; Villablanca, J. P.; Viñuela, F.; Feng, L.; Loh, Y.; Liebeskind, D. S., Mechanical thrombectomy for acute ischemic stroke with cerebral microbleeds. *J Neurointerv Surg* **2016,** *8* (6), 563-567.

53. Bivard, A.; Cheng, X.; Lin, L. T.; Levi, C.; Spratt, N.; Kleinig, T.; O'Brien, B.; Butcher, K.; Lou, M.; Zhang, J. F.; Sylaja, P. N.; Cao, W. J.; Jannes, J.; Dong, Q.; Parsons, M., Global white matter hypoperfusion on CT predicts larger infarcts and hemorrhagic transformation after acute ischemia. *CNS Neurosci Ther* **2016,** *22* (3), 238-243.

54. Cappellari, M.; Carletti, M.; Danese, A.; Bovi, P., Early introduction of direct oral anticoagulants in cardioembolic stroke patients with non-valvular atrial fibrillation. *J Thromb Thrombolysis* **2016,** *42* (3), 393-398.

55. Purrucker, J. C.; Wolf, M.; Haas, K.; Rizos, T.; Khan, S.; Dziewas, R.; Kleinschnitz, C.; Binder, A.; Gröschel, K.; Hennerici, M. G.; Lobotesis, K.; Poli, S.; Seidel, G.; Neumann-Haefelin, T.; Ringleb, P. A.; Heuschmann, P. U.; Veltkamp, R., Safety of endovascular thrombectomy in patients receiving non-nitamin K antagonist oral anticoagulants. *Stroke* **2016,** *47* (4), 1127-1130.

56. Gioia, L. C.; Kate, M.; Sivakumar, L.; Hussain, D.; Kalashyan, H.; Buck, B.; Bussiere, M.; Jeerakathil, T.; Shuaib, A.; Emery, D.; Butcher, K., Early rivaroxaban use after cardioembolic stroke may not result in hemorrhagic transformation: a prospective magnetic resonance imaging study. *Stroke* **2016,** *47* (7), 1917-1919.

57. Heo, J. H.; Song, D.; Nam, H. S.; Kim, E. Y.; Kim, Y. D.; Lee, K. Y.; Lee, K. J.; Yoo, J.; Kim, Y. N.; Lee, B. C.; Yoon, B. W.; Kim, J. S., Effect and safety of rosuvastatin in acute ischemic stroke. *J Stroke* **2016,** *18* (1), 87-95.

58. Liu, J.; Wang, D.; Li, J.; Xiong, Y.; Liu, B.; Wei, C.; Wu, S.; Lin, J.; Liu, M., Increased serum alkaline phosphatase as a predictor of symptomatic hemorrhagic transformation in ischemic stroke patients with atrial fibrillation and/or rheumatic heart disease. *J Stroke Cerebrovasc Dis* **2016,** *25* (10), 2448-2452.

59. Ho, B. L.; Chen, C. F.; Lin, R. T.; Liu, C. K.; Chao, A. C., Clinical implication of hemorrhagic transformation in ischemic stroke patients treated with recombinant tissue plasminogen activator. *Neurol Sci* **2016,** *37* (11), 1799-1805.

60. Guo, Z.; Yu, S.; Xiao, L.; Chen, X.; Ye, R.; Zheng, P.; Dai, Q.; Sun, W.; Zhou, C.; Wang, S.; Zhu, W.; Liu, X., Dynamic change of neutrophil to lymphocyte ratio and hemorrhagic transformation after thrombolysis in stroke. *J Neuroinflammation* **2016,** *13* (1), 199.

61. Bas, D. F.; Ozdemir, A. O.; Colak, E.; Kebapci, N., Higher insulin resistance level is associated with worse clinical response in acute ischemic stroke patients treated with intravenous thrombolysis. *Transl Stroke Res* **2016,** *7* (3), 167-171.

62. Chen, X.; Wang, Y.; Fu, M.; Lei, H.; Cheng, Q.; Zhang, X., Plasma immunoproteasome predicts early hemorrhagic transformation in acute ischemic stroke patients. *J Stroke Cerebrovasc Dis* **2017,** *26* (1), 49-56.

63. Wei, C. C.; Zhang, S. T.; Wang, Y. H.; Liu, J. F.; Li, J.; Yuan, R. Z.; Tan, G.; Zhang, S. H.; Liu, M., Association between leukoaraiosis and hemorrhagic transformation after cardioembolic stroke due to atrial fibrillation and/or rheumatic heart disease. *J Neurol Sci* **2017,** *378*, 94-99.

64. Castro, P.; Azevedo, E.; Serrador, J.; Rocha, I.; Sorond, F., Hemorrhagic transformation and cerebral edema in acute ischemic stroke: Link to cerebral autoregulation. *J Neurol Sci* **2017,** *372*, 256-261.

65. Purrucker, J. C.; Haas, K.; Wolf, M.; Rizos, T.; Khan, S.; Kraft, P.; Poli, S.; Dziewas, R.; Meyne, J.; Palm, F.; Jander, S.; Möhlenbruch, M.; Heuschmann, P. U.; Veltkamp, R., Haemorrhagic transformation after ischaemic stroke in patients taking non-vitamin K antagonist oral anticoagulants. *J Stroke* **2017,** *19* (1), 67-76.
